# Helminth co-infections have no additive detrimental impact on milk yield and milk quality compared to mono-infections in German dairy cows

**DOI:** 10.1186/s13071-024-06470-8

**Published:** 2024-09-19

**Authors:** Katharina May, Anna Sophie Hecker, Sven König, Christina Strube

**Affiliations:** 1 Institute of Animal Breeding and Genetics, Justus-Liebig-University of Gießen, 35390 Giessen, Germany; 2 Institute for Parasitology, Centre for Infection Medicine, University of Veterinary Medicine Hannover, Buenteweg 17, 30559 Hannover, Germany

**Keywords:** *Fasciola hepatica*, Liver flukes, Rumen flukes, Gastrointestinal nematodes, Trichostrongyles, *Ostertagia ostertagi*, Lungworms, *Dictyocaulus viviparus*, Performance, Cattle

## Abstract

**Background:**

Infections with (tricho-)strongyles, *Dictyocaulus viviparus* or *Fasciola hepatica* have been shown to reduce milk production in dairy cows. However, the current published studies focused on one single helminth infection by neglecting helminth co-infections and their possible (additive) effects on host performance. Hence, for the first time, we investigated differences in the impact of patent helminth co-infections versus mono-infections on milk production parameters in individual cows.

**Methods:**

A total of 1583 dairy cows from 27 herds were included in this study. Faecal samples were examined in 2015 and 2021/2022 to determine the number of eggs/larvae per gram faeces for (tricho-)strongyles, *D. viviparus*, *F. hepatica* and rumen flukes. The cows were classified as non-infected, mono-infected and co-infected. Linear mixed models were applied to analyse the association between infection status (non-infected *vs.* mono-infected *vs.* co-infected) with milk yield, milk protein and milk fat content by including potential confounders.

**Results:**

Infections with (tricho-)strongyles, *D. viviparus*, *F. hepatica* and rumen flukes were detected in 100%, 28.6%, 50.0% and 21.4% of herds, and 27.4%, 2.6%, 10.8% and 0.8% of faecal samples in 2015, while 100%, 0.0%, 86.7% and 60.0% of herds and 52.3%, 0.0%, 13.3% and 26.8% of faecal samples were positive in 2021/2022. Co-infections with two or more helminth taxa were detected in 74.4% of herds and 5.0% of faecal samples in 2015, and in 93.3% of herds and 21.7% of faecal samples in 2021/2022. The correlations between strongyle EPG, *D. viviparus* LPG and *F. hepatica* EPG were significantly positive in 2015. Significantly higher mean EPGs were identified in 2015 in faecal samples presenting co-infections with *F. hepatica* and one or two other helminth taxa than in faecal samples presenting *F. hepatica* mono-infections (*P* = 0.013). Although expected, the infection status (mono- or co-infected) had no significant impact on milk yield, milk protein and milk fat content in the linear mixed model analyses based on individual faecal examinations.

**Conclusions:**

Patent helminth co-infections had no additive detrimental impact on milk production parameters in the present study. This might be a result of presumably low worm burdens, but should be confirmed in future studies.

**Graphical Abstract:**

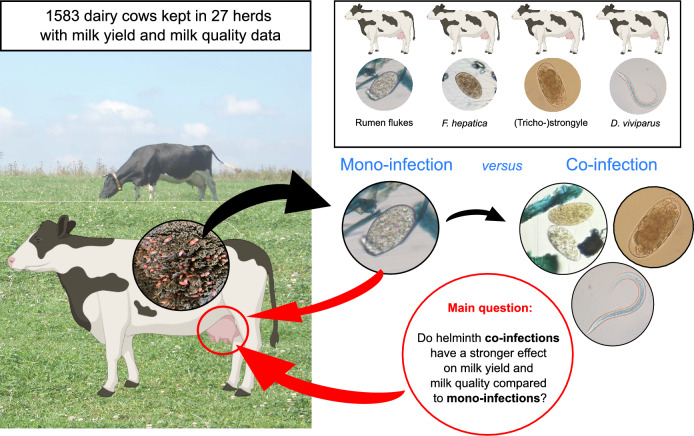

**Supplementary Information:**

The online version contains supplementary material available at 10.1186/s13071-024-06470-8.

## Background

In Europe, the most economically relevant helminth infections in dairy cows comprise gastrointestinal nematodes (GIN; mainly the trichostrongyle *Ostertagia ostertagi*), the bovine lungworm (*Dictyocaulus viviparus*) and the liver fluke (*Fasciola hepatica*) [1]. The impact of infections on milk yield and milk quality in dairy cows on an individual animal level was investigated comprehensively for each helminth infection separately. Reported milk production losses due to *O. ostertagi*, *D. viviparus* or *F. hepatica* infections, as usually determined by faecal examinations or antibody levels in individual cows, ranged from 1.9 to 15.0% in affected herds [1, 2]. Moreover, significant negative associations between increased *O. ostertagi* bulk-tank milk (BTM) antibody levels or patent GIN infections in individual cows with milk yield and milk protein content have been reported [3–5]. In addition, Charlier et al. [6] associated an increase in *F. hepatica*-BTM antibody levels with a significant 0.06% decrease in milk fat content. A further seroprevalence study reported a significant decrease of − 1.62 kg milk per cow per day and − 0.06 kg milk protein and fat yield in *F. hepatica* strongly infected herds compared to non-infected herds [7]. Another study showed a significant decrease of 0.02% in annual average milk protein and fat content in *D. viviparus*-seropositive compared to seronegative herds [8].

However, the main limitation of the current published studies arises from the fact that the impact of helminth infections on milk production in dairy cows was examined with a sole focus on a single parasite taxon (GIN or *D. viviparus* or *F. hepatica*). This may have led to bias or overestimated economic losses in a number of studies, since co-infections were not always ruled out. Springer et al. [5] analysed patterns of dairy cow helminth co-exposure based on 646 BTM samples from Germany. Depending on the geographical region, they identified co-exposure to *O. ostertagi* and *D. viviparus* in up to 4.5% of herds, co-exposure to *O. ostertagi* and *F. hepatica* in up to 22.4%, and co-exposure to all three helminth taxa in up to 2.0% of herds. However, antibody levels circulate in the host even after the infection has been cleared. Thus, measured antibody levels do not represent a reliable parameter to actually assess current helminth co-infections, but large-scale studies based on individual faecal examinations are capable of estimating the status of patent co-infections and its effect on dairy cow performance.

Jones et al. [9] found 46.0% of cattle herds in Wales, United Kingdom (UK), to be co-infected with *F. hepatica* and rumen flukes based on faecal examinations. Similarly, Sargison et al. [10] showed that 45.0% of the slaughtered beef cattle were co-infected with these flukes in the UK. The impact of simultaneous infection with *F. hepatica* and rumen flukes on cattle health and production needs further research since the rumen fluke prevalence rapidly increased in western Europe in recent years [11]. Rumen fluke infections are usually subclinical, but can lead to severe diarrhoea and even death in infected cattle [12]. However, the impact of patent rumen fluke infections on milk yield and milk quality in dairy cows is currently unknown. Only one published study reported a significant increase in milk yield in dairy cows infected with GIN and paramphistomes after anthelmintic treatment [13]. Patent rumen fluke and *F. hepatica* co-infections in dairy cows were detected by May et al. [14], but association analyses between co-infections and milk production parameters were not conducted, as the rumen fluke prevalence was below 1.0% in the German dairy herds studied in 2015.

Regarding the impact of helminth co-infections on host production, Charlier et al. [6] studied the effect of co-exposure to *F. hepatica* and *O. ostertagi* on milk yield in Belgian dairy herds by including BTM antibody levels from both helminth species as an interaction term in the statistical model. The interaction term was not statistically significant in the model, i.e., herds with high BTM antibody levels for both *F. hepatica* and *O. ostertagi* had no increased risk for milk production losses compared to negative herds or those with high BTM antibody levels for only one helminth species. Another study based on herd BTM antibody levels categorised German dairy herds into parasite-free, *O. ostertagi* mono-infected, *F. hepatica* mono-infected and co-infected, and found a lower chance of exposure to both parasites in herds with higher milk yield [15]. Springer et al. [5] identified a significantly increased proportion of cows in suboptimal body condition score in German dairy herds showing positive BTM ELISA results for both *O. ostertagi* and *F. hepatica* compared to seronegative herds or those being positive for only one helminth species.

Based on these preliminary studies, we hypothesize that co-infections might have a stronger impact on dairy cow performance compared to mono-infections. Therefore, the objective of this study was to analyse the impact of helminth co-infections on milk yield, milk protein and milk fat content in two different datasets of German dairy cows with individual faecal examinations. In this context, we also aimed to add data on the impact of patent rumen fluke infections on milk production parameters in dairy cows.

## Methods

### Dairy herds and datasets

A total number of 27 dairy herds and 1583 dairy cows corresponding to two different datasets were included in this study.

Dataset 1 (DS1) included 922 pastured dairy cows from 14 (10 organic and four conventional) German dairy herds (Table 1) located in the federal states of Hesse (two herds), North Rhine-Westphalia (three herds), Lower Saxony (eight herds) and Schleswig–Holstein (one herd). The DS1 cows represent a subset of a dataset previously used to study associations between GIN, *D. viviparus* and *F. hepatica* with milk production and fertility parameters without considering potential co-infections [5, 14, 16]. The herds and cows in DS1 were part of a “pasture genetics project” aiming at the comparison of helminth infections in different breeds and genetic lines [17]. All DS1 cows had access to pasture for more than 8 h per day from April/May to November 2015 and were not treated with anthelmintics in the sampling year. Faecal samples were collected rectally from all milking cows in each herd in July and September 2015 to detect helminth infections, resulting in 1524 faecal samples for DS1. Faecal samples from both July and September 2015 were available for 602 cows. The average number of faecal samples per cow was 1.7. The total number of examined cows in DS1 herds ranged from 26 to 195 with a mean of 65.9 cows.

**Table 1:** Descriptive statistics for the characteristics of the two datasets (DS1 and DS2) and for milk production parameters used in statistical model analyses

Parameter	Dataset 1 (DS1)						Dataset 2 (DS2)					
	No. of cows (%)	No. of records (%)	Mean	SD	Min	Max	No. of cows (%)	No. of records (%)	Mean	SD	Min	Max
Breed												
DSN	328/922 (35.6)						60/667 (9.0)					
GH	594/922 (64.4)						445/667 (66.7)					
GH crossbred	–						162/667 (24.3)					
Housing system												
Conventional	377/922 (40.9)						490/667 (73.5)					
Organic	545/922 (59.1)						177/667 (26.5)					
Season^a^												
Spring		–					48/667 (7.2)					
Summer		792/1524 (52.0)					273/667 (40.9)					
Autumn		732/1524 (48.0)					275/667 (41.2)					
Winter		–					71/667 (10.6)					
Milk production												
MY (kg)	922	1524	19.60	7.02	5.10	40.50	667	667	25.34	8.44	3.00	53.60
P%	922	1524	3.28	0.66	0.87	4.95	667	667	3.50	0.38	2.56	4.92
F%	922	1524	4.05	0.92	1.16	6.58	667	667	4.13	0.61	2.55	6.11
Parity number^b^	922	1524	2.64	1.41	1.00	5.00	667	667	2.62	1.43	1.00	5.00
Days in milk^c^	922	1524	188.57	116.01	5.00	561.00	667	667	188.47	120.27	5.00	514.00

DSN: German Black Pied cattle (*Deutsches Schwarzbuntes Niederungsrind*); GH: German Holstein; MY: milk yield (in kg); P%: protein percentage; F%: fat percentage; SD: standard deviation

^a^Season: autumn = September, October, November; spring = March, April, May; summer = June, July, August; winter = December, January, February. Note that only one herd was examined in spring (May), and only two herds in winter (February) in DS2

^b^Cows with parity > 4 were classified as parity = 5

^c^Days in milk at time of first milk production test-day record after the faecal examination for DS1 and average days in milk from both milk production test-day records before and after the faecal examination, or days in milk at one test-day milk production record in case of only one available test-day record

Dataset 2 (DS2) included 667 pastured dairy cows from 15 (four organic and 11 conventional) German dairy herds (Table 1) located in the federal states of Hesse (one herd) and Lower Saxony (14 herds). The DS2 cows represent a subset of a cow dataset previously used to study in-herd prevalences and to compare two coproscopical methods for *F. hepatica* and rumen fluke infections in German dairy cows [18]. The selected herds, representing two breeds, participated in a project starting in May 2021 and aiming at genetic mechanisms for resistance against *F. hepatica* in dairy cows. The selection of DS2 herds was based on i) patent *F. hepatica* infections in 2020, or ii) *F. hepatica*-positive BTM samples in 2020 assessed in the laboratory of the state control association for milk recording (*Landeskontrollverband Weser-Ems, Leer, Germany*) with the IDEXX Fasciolosis Verification test ELISA kit (Montpellier, France). Faecal samples were collected rectally once from approximately 50 randomly selected milking cows per herd between May 2021 and February 2022. The total number of examined cows in the DS2 herds ranged from 33 to 52 with a mean of 44.5 cows. In four herds, anthelmintic treatment (closantel, triclabendazole or oxyclozanide) was routinely applied in dry cows.

**Fig. 1:**
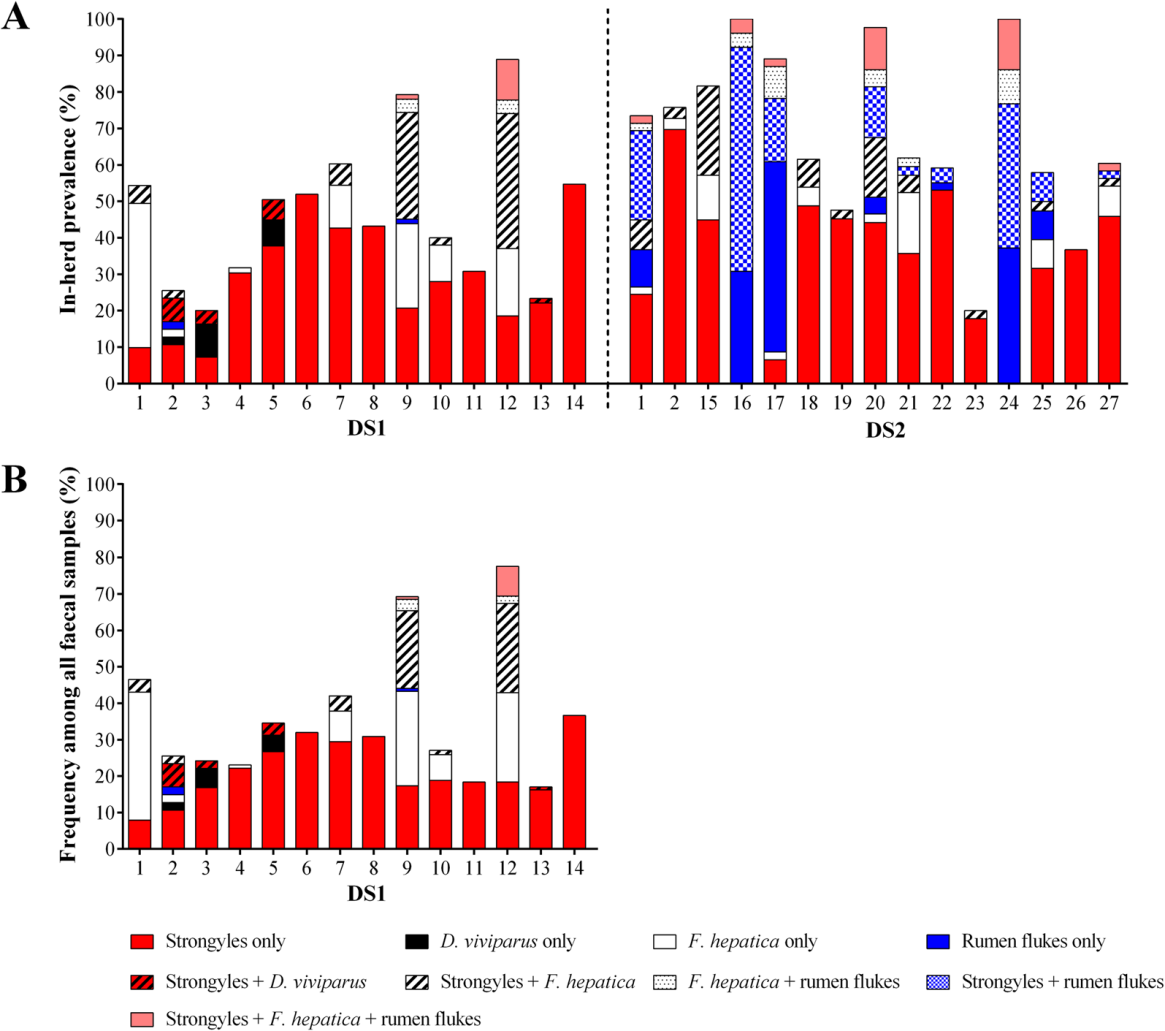
In-herd prevalence of patent helminth mono- and co-infections in the two datasets (DS1, sampled in 2015, and DS2, sampled in 2021/2022) (**A**) and frequency of positive faecal samples in the repeatedly sampled DS1 (**B**). Note that herd no. 1and 2 were included in both datasets

Two herds (herd no. 1 and 2, cf. Figure 1) were visited in 2015 and in 2021/2022 and were therefore included in both datasets.

Descriptive statistics for breed, housing system and season of faecal sampling of the two datasets are presented in Table 1.

### Faecal examinations of individual cows

For the cows in DS1, the number of strongyle eggs per gram faeces (EPG) was examined with a modified McMaster technique using 4 g faeces and saturated NaCl solution as flotation medium according to Thienpont et al. [19]. The analytical sensitivity was 25 EPG. For the DS2 cows, strongyle EPGs were specified with the FLOTAC double technique using 10 g faeces and zinc sulfate solution as flotation medium [20]. The analytical sensitivity was 2 EPG. *Fasciola hepatica* and rumen fluke EPGs were determined with the sedimentation technique using 10 g faeces. The number of *D. viviparus* larvae per gram faeces (LPG) was determined with the Baermann method by loading Baermann funnels with 40 g faeces at the day of sampling and microscopic examination approximately 18 h later.

### Milk production data

Individual cow data (e.g., milk production, parity, lactation stage) were provided by the National Genetic Evaluation Center (*Vereinigte Informationssysteme Tierhaltung w.V., VIT*). Test-day milk production data (i.e., test-day records) included the monthly recorded milk yield (MY [kg milk/cow/day]), milk protein content (P%) and milk fat content (F%).

For both DS1 and DS2, we generated different datasets to combine the test-day milk production data (i.e., one milk production record per cow per month) of individual cows with the results of faecal examinations prior to the linear mixed model analyses to test the effect of the cow’s infection status on milk production parameters. These datasets included the mean of two test-day records (i.e., the mean of two months with one record per cow per month) after the faecal examination, the mean of three test-day records (i.e., the mean of three months with one record per cow per month) after the faecal examination, the mean of both test-day records within 30 days before and 30 days after the faecal examination (i.e., the mean of two months with one record per cow per month), and the mean of all test-day records within 40 days before and 40 days after the faecal examination (i.e., up to four records per cow with one record per cow per month). Afterwards, we compared Akaike information criteria (AIC) values from the models for all four generated milk production datasets within DS1 and within DS2. This procedure was applied to select the optimal time period of milk production test-day recording before and after faecal examination for the linear mixed model analyses. For the selection of the time periods between faecal examination and milk production test-day recording, we aimed to include records close to the time of the faecal examination to ensure a reliable association with the observed infection status, and at the same time to increase the number of test-day records per cow in order to improve model quality.

Descriptive statistics for test-day milk production data of the two datasets finally used in linear mixed model analyses is given in Table 1.

### Definition of infection status

In both datasets, a cow was binary classified as either ‘infected’ (EPG/LPG > 0) or ‘non-infected’ (EPG/LPG = 0) for examined helminth infection (strongyles, *D. viviparus*, *F. hepatica*, rumen flukes). The DS1 cows were classified as ‘non-infected’, ‘mono-infected’ or ‘co-infected’ for the respective faecal examination in July 2015 and September 2015. Thus, in case of repeated sampling in July and September, cows were classified separately for each faecal sampling. In this dataset, cows were classified only independent of the helminth taxon detected, i.e. a cow was classified as mono-infected in case of a single infection with any of the taxa, and as co-infected in case of infection with two or more helminth taxa (Table 2). A taxon-related classification as applied for DS2 was not possible for the DS1 cows since the number of cows with rumen fluke and *D. viviparus* mono-infections (0.1% and 0.9%, respectively) was too low for the following statistical model analyses.

**Table 2:** Infection status as used in the statistical model (1) derived from 1524 individual faecal samples of 922 cows in dataset (DS) 1, and infection status (classification 1 and classification 2) as used in the statistical model (2) from the 667 faecal samples of 667 cows in DS2

Infection status	Description	No. (%) of faecal samples
DS1		
Non-infected	Negative for all helminth taxa (strongyles, *D. viviparus*, *F. hepatica* and rumen flukes)	972 (63.8%)
Mono-infected	Infected with only one helminth taxon (strongyles, *D. viviparus*, *F. hepatica* or rumen flukes)	475 (31.2%)
Co-infected	Infected with two or more helminth taxa (strongyles/*D. viviparus*/*F. hepatica*/rumen flukes)	77 (5.0%)
DS2		
Classification 1		
Non-infected	Negative for all helminth taxa (strongyles, *F. hepatica* and rumen flukes)	211 (31.6%)
Mono-infected	Infected with only one helminth taxon (strongyles, *F. hepatica* or rumen flukes)	311 (46.6%)
Co-infected	Infected with two or more helminth taxa (strongyles/*F. hepatica*/rumen flukes)	145 (21.8%)
Classification 2		
Non-infected	Negative for all helminth taxa (strongyles, *F. hepatica* and rumen flukes)	211 (31.6%)
Mono-infection status 1	Strongyle positive, *F. hepatica* and rumen fluke negative	218 (32.7%)
Mono-infection status 2	*F. hepatica* positive, strongyle and rumen fluke negative	26 (3.9%)
Mono-infection status 3	Rumen fluke positive, strongyle and *F. hepatica* negative	67 (10.0%)
Co-infection status 1	Strongyle and *F. hepatica* positive, rumen fluke negative	33 (5.0%)
Co-infection status 2	Strongyle and rumen fluke positive, *F. hepatica* negative	82 (12.3%)
Co-infection status 3	*F. hepatica* and rumen fluke positive, strongyle negative	14 (2.1%)
Co-infection status 4	Strongyle, *F. hepatica* and rumen fluke positive	16 (2.4%)

The same classification was applied for DS2 cows, all of which were sampled only once (Table 2, classification 1). Additionally, DS2 cows were classified with regard to the helminth taxa detected (Table 2, classification 2). Since all cows in DS2 were negative for *D. viviparus* infections, the classification was based only on strongyle, *F. hepatica* and rumen fluke infections.

### Data analyses

All statistical analyses were performed by use of the software SAS® OnDemand for Academics (SAS Institute Inc. 2014; Cary, NC, USA). The PROC FREQ and PROC MEANS procedures were used for all descriptive statistics, and to calculate the EPG/LPG interquartile range (25th to 75th percentile).

#### Descriptive statistics

Correlations between EPGs and/or LPGs for different helminth taxa were analysed using Pearson’s correlation including positive and negative EPG/LPG counts. Differences in the mean rank of EPGs/LPGs between mono- and co-infected cows in each dataset were tested via Mann–Whitney test (e.g., *F. hepatica* EPG in mono-infected samples was tested against *F. hepatica* EPG in samples co-infected with one or more other helminth taxa). *P* ≤ 0.05 were regarded statistically significant for Pearson correlation analyses and the Mann–Whitney test.

#### Linear mixed models

Linear mixed models were used to analyse the relationship between patent helminth infections and milk production parameters.

Linear mixed model (1) applied on DS1 was defined to analyse the association between the independent variable infection status (co-infected *vs*. mono-infected *vs*. non-infected) and mean test-day milk production parameters milk yield (MY-DS1), milk protein content (P%-DS1) and milk fat content (F%-DS1) as dependent variables. Further fixed effects included were the herd (14 herds), genetic line (DSN, GHm, GHp, GHnz), parity number (1, 2, 3, 4, > 4), lactation stage, i.e. days in milk (DIM) at time of first test-day after the faecal examination (five classes: DIM ≤ 14; DIM 15 to 77; DIM 78 to 140; DIM 141 to 231; DIM ≥ 232), the season of milk production recording (summer and autumn), and a combination of calving year and calving month (eight classes: year 2014 or 2015 combined with the four different seasons [January–March, April–June, July–September, October–December]). The cow (with up to two faecal examinations per cow) was modelled as a random effect. Only cows with at least two test-day records after each faecal examination were included in the analysis. As parity number, genetic line and season of milk production recording were not statistically significant for P%-DS1, and parity number was not statistically significant for F%-DS1, these effects were removed from the model for P%-DS1 and F%-DS1. The interactions between infection status and herd as well as infection status and faecal sampling period (July or September) were tested but not statistically significant, and thus not considered in model 1.

Linear mixed model (2) applied on DS2 was defined to analyse the association between the independent variable infection status (co-infected *vs*. mono-infected *vs*. non-infected according to classification 1 or classification 2, respectively) and mean test-day milk production parameters milk yield (MY-DS2), milk protein content (P%-DS2) and milk fat content (F%-DS2 as dependent variables. For cows with only one available test-day record within 30 days before or after faecal examination, we included this one test-day record in the model analysis. The herd (15 herds), parity number (1, 2, 3, 4, > 4) and a combination of calving year and calving season (eight classes: year 2020 or 2021 combined with the four different seasons [January-March, April-June, July–September, October-December]) were included as further independent variables and modelled as fixed effects. Days in milk (average DIM from both test-day records before and after the faecal examination or DIM at one test-day record in case of only one available test-day) was included as a covariate (linear regression). The season of milk production recording was not included as an effect in model 2, because the model did not converge due to an overlap of herd and seasonal effect (i.e., only one visit per herd). In accordance to model 1, we included the interactions between infection status (classification 1 and classification 2, respectively) and herd as well as between infection status and month of faecal sampling in model 2, but the interaction terms were not statistically significant and therefore removed from the model. The number of observations within effect classes for model 1 and 2 are given in Additional file 1: Table S1–S3.

#### Type III test statistics and least-squares means

We estimated least-squares means (LSM) for all milk production parameters in linear mixed models 1 and 2, and we tested the significance of fixed effects and covariates (linear regression) via *F*-tests (sum of squares type III test statistics = overall *F*-test). We selected the model with the smallest AIC for each dependent variable as the final model. For model validation, the normal distribution of residuals and random variances was checked by quantile–quantile plots and by Shapiro–Wilk tests. Pairwise differences of LSM for infection status in model 1 and 2 were estimated by applying the Bonferroni adjustment for multiple testing correction. *P* ≤ 0.05 were regarded as statistically significant for the overall *F*-tests and for the (adjusted) pairwise differences of LSM.

## Results

### Frequency of helminth taxa on herd and in-herd level

The in-herd prevalence in the 27 different herds and the frequency of patent helminth mono- and co-infections in the two datasets are summarised in Fig. 1. Patent strongyle (Trichostrongylidae and other Strongylida) infections were present in all herds. Patent infections with *D. viviparus* were only detected in 14.8% (4/27) of herds (herd no. 2, 3, 5 and 13), all belonging to DS1. *Fasciola hepatica* and rumen fluke eggs were identified in 66.7% (18/27) and 44.4% (12/27) of herds, respectively. Detailed data on in-herd prevalence and mean EPG/LPG among positive faecal samples of the individual herds in the two datasets are presented in Table 3 (strongyles and lungworms) and Table 4 (flukes). Corresponding data on mean EPG/LPG among all faecal samples (positives and negatives) of the individual herds are presented in Additional file 2: Table S1, Table S2.

**Table 3:** In-herd prevalence for strongyles and *D. viviparus* and mean egg or larvae count per gram faeces (EPG/LPG) among positive faecal samples with corresponding standard deviation (SD), range (minimum and maximum), median and interquartile range (25th to 75th percentile) of the dairy herds in dataset 1 (DS1) and dataset 2 (DS2)

Herd (no.)	Strongyles				*D. viviparus*			
	Positive faecal samples (%)	Mean EPG ± SD (range)	Median	Interquartile range	Positive faecal samples (%)	Mean LPG ± SD (range)	Median	Interquartile range
Dataset 1 (DS1)								
1	12/81 (14.8)	32.7 ± 15.8 (25.0–75.0)	25.0	25.0–25.0	0/81 (0.0)	0.0	0.0	0.0–0.0
2	9/47 (19.2)	38.9 ± 33.3 (25.0–125.0)	25.0	25.0–25.0	4/47 (8.5)	0.2 ± 0.2 (0.1–0.5)	0.1	0.1–0.3
3	17/55 (30.9)	31.9 ± 11.5 (25.0–50.0)	25.0	25.0–50.0	7/55 (12.7)	0.1 ± 0.04 (0.03–0.13)	0.1	0.03–0.08
4	20/66 (30.3)	48.1 ± 32.3 (25.0–150.0)	37.5	25.0–50.0	0/66 (0.0)	0.0	0.0	0.0–0.0
5	85/196 (43.4)	47.2 ± 33.9 (25.0–225.0)	25.0	25.0–50.0	25/196 (12.8)	0.3 ± 0.4 (0.03–1.15)	0.1	0.03–0.5
6	27/52 (51.9)	35.0 ± 18.1 (25.0–100.0)	25.0	25.0–50.0	0/52 (0.0)	0.0	0.0	0.0–0.0
7	33/68 (48.5)	38.1 ± 25.3 (25.0–125.0)	25.0	25.0–50.0	0/68 (0.0)	0.0	0.0	0.0–0.0
8	19/44 (43.2)	38.1 ± 20.3 (25.0–100.0)	25.0	25.0–50.0	0/44 (0.0)	0.0	0.0	0.0–0.0
9	42/82 (51.2)	39.2 ± 19.5 (25.0–75.0)	25.0	25.0–50.0	0/82 (0.0)	0.0	0.0	0.0–0.0
10	15/48 (31.2)	33.8 ± 15.2 (25.0–75.0)	25.0	25.0–50.0	0/48 (0.0)	0.0	0.0	0.0–0.0
11	8/26 (30.8)	33.3 ± 17.7 (25.0–75.0)	25.0	25.0–25.0	0/26 (0.0)	0.0	0.0	0.0–0.0
12	18/27 (66.7)	51.0 ± 29.6 (25.0–125.0)	50.0	25.0–50.0	0/27 (0.0)	0.0	0.0	0.0–0.0
13	18/77 (23.4)	30.7 ± 13.2 (25.0–75.0)	25.0	25.0–25.0	1/77 (1.3)	0.03^a^	0.03	0.03–0.03
14	29/53 (54.7)	44.6 ± 22.2 (25.0–100.0)	50.0	25.0–50.0	0/53 (0.0)	0.0	0.0	0.0–0.0
Total	352/922 (38.2)	41.1 ± 25.9 (25.0–225.0)	25.0	25.0–50.0	37/922 (4.0)	0.2 ± 0.3 (0.03–1.2)	0.08	0.03–0.15
Dataset 2 (DS2)								
1	29/49 (59.2)	4.8 ± 4.7 (2.0–24.0)	2.0	2.0–6.0	0/49 (0.0)	0.0	0.0	0.0–0.0
2	24/33 (72.7)	3.3 ± 2.3 (2.0–10.0)	2.0	2.0–4.0	0/33 (0.0)	0.0	0.0	0.0–0.0
15	34/49 (69.4)	6.6 ± 6.4 (2.0–22.0)	4.0	2.0–10.0	0/49 (0.0)	0.0	0.0	0.0–0.0
16	34/52 (65.4)	5.7 ± 5.0 (2.0–26.0)	4.0	2.0–6.0	0/52 (0.0)	0.0	0.0	0.0–0.0
17	12/46 (26.1)	7.2 ± 7.3 (2.0–24.0)	4.0	2.0–8.0	0/46 (0.0)	0.0	0.0	0.0–0.0
18	22/39 (56.4)	5.5 ± 5.4 (2.0–20.0)	2.0	2.0–8.0	0/39 (0.0)	0.0	0.0	0.0–0.0
19	20/42 (47.6)	4.6 ± 3.1 (2.0–12.0)	4.0	2.0–7.0	0/42 (0.0)	0.0	0.0	0.0–0.0
20	37/43 (86.1)	9.4 ± 14.1 (2.0–84.0)	6.0	4.0–8.0	0/43 (0.0)	0.0	0.0	0.0–0.0
21	19/42 (45.2)	4.6 ± 6.8 (2.0–32.0)	2.0	2.0–4.0	0/42 (0.0)	0.0	0.0	0.0–0.0
22	28/49 (57.1)	10.8 ± 15.1 (2.0–52.0)	4.0	2.5–8.0	0/49 (0.0)	0.0	0.0	0.0–0.0
23	9/49 (20.0)	2.7 ± 1.4 (2.0–6.0)	2.0	2.0–2.0	0/49 (0.0)	0.0	0.0	0.0–0.0
24	23/43 (53.5)	10.0 ± 13.2 (2.0–54.0)	4.0	2.0–12.0	0/43 (0.0)	0.0	0.0	0.0–0.0
25	16/38 (42.1)	5.4 ± 5.4 (2.0–20.0)	3.0	2.0–7.0	0/38 (0.0)	0.0	0.0	0.0–0.0
26	18/49 (36.7)	8.8 ± 12.3 (2.0–54.0)	5.0	4.0–8.0	0/49 (0.0)	0.0	0.0	0.0–0.0
27	24/48 (52.1)	6.4 ± 6.6 (2.0–32.0)	4.0	2.0–10.0	0/48 (0.0)	0.0	0.0	0.0–0.0
Total	349/667 (52.3)	6.7 ± 9.0 (2.0–84.0)	4.0	2.0–8.0	0/667 (0.0)	0.0	0.0	0.0–0.0

^a^Standard deviation and range are not given as only one sample was positive

**Table 4:** In-herd prevalence for *F. hepatica* and rumen flukes and mean egg count per gram faeces (EPG) among positive faecal samples with corresponding standard deviation (SD), range (minimum and maximum), median and interquartile range (25th to 75th percentile) of the dairy herds in dataset 1 (DS1) and dataset 2 (DS2)

Herd (no.)	*F. hepatica*				Rumen flukes			
	Positive faecal samples (%)	Mean EPG ± SD (range)	Median	Interquartile range	Positive faecal samples (%)	Mean EPG ± SD (range)	Median	Interquartile range
Dataset 1 (DS1)								
1	36/81 (44.4)	0.4 ± 0.4 (0.1–1.6)	0.2	0.1–0.5	0/81 (0.0)	0.0	0.0	0.0–0.0
2	2/47 (4.3)	0.3 ± 0.1 (0.2–0.3)	0.3	0.2–0.3	1/47 (2.1)	0.9^a^	0.9	0.9–0.9
3	0/55 (0.0)	0.0	0.0	0.0–0.0	0/55 (0.0)	0.0	0.0	0.0–0.0
4	1/66 (1.5)	0.2^a^	0.2	0.2–0.2	0/66 (0.0)	0.0	0.0	0.0–0.0
5	0/196 (0.0)	0.0	0.0	0.0–0.0	0/196 (0.0)	0.0	0.0	0.0–0.0
6	0/52 (0.0)	0.0	0.0	0.0–0.0	0/52 (0.0)	0.0	0.0	0.0–0.0
7	12/68 (17.6)	0.8 ± 0.8 (0.1–2.4)	0.3	0.1–1.4	0/68 (0.0)	0.0	0.0	0.0–0.0
8	0/44 (0.0)	0.0	0.0	0.0–0.0	0/44 (0.0)	0.0	0.0	0.0–0.0
9	46/82 (56.1)	0.5 ± 0.9 (0.1–7.1)	0.3	0.1–0.5	5/82 (6.1)	0.3 ± 0.2 (0.1–0.5)	0.2	0.1–0.4
10	6/48 (12.5)	0.3 ± 0.3 (0.1–0.8)	0.1	0.1–0.4	0/48 (0.0)	0.0	0.0	0.0–0.0
11	0/26 (0.0)	0.0	0.0	0.0–0.0	0/26 (0.0)	0.0	0.0	0.0–0.0
12	19/27 (70.4)	1.5 ± 1.8 (0.1–8.9)	0.3	0.4–2.1	4/27 (14.8)	0.3 ± 0.3 (0.1–0.7)	0.2	0.1–0.2
13	0/77 (0.0)	0.0	0.0	0.0–0.0	0/77 (0.0)	0.0	0.0	0.0–0.0
14	0/53 (0.0)	0.0	0.0	0.0–0.0	0/53 (0.0)	0.0	0.0	0.0–0.0
Total	122/922 (13.2)	0.7 ± 1.1 (0.1–8.9)	0.3	0.1–0.8	10/922 (1.1)	0.3 ± 0.3 (0.1–0.9)	0.2	0.1–0.5
Dataset 2 (DS2)								
1	7/49 (14.3)	0.2 ± 0.1 (0.1–0.5)	0.1	0.1–0.2	19/49 (38.8)	0.8 ± 1.3 (0.1–4.8)	0.2	0.2–0.6
2	2/33 (6.1)	0.2 ± 0.1 (0.1–0.3)	0.2	0.1–0.3	0/33 (0.0)	0.0	0.0	0.0–0.0
15	18/49 (36.7)	0.7 ± 0.7 (0.1–2.5)	0.3	0.2–0.9	0/49 (0.0)	0.0	0.0	0.0–0.0
16	4/52 (7.7)	0.1 ± 0.0 (0.1–0.1)	0.1	0.1–0.1	52/52 (100.0)	33.8 ± 42.8 (0.3–192.7)	18.5	7.5–41.6
17	6/46 (13.0)	0.2 ± 0.2 (0.1–0.5)	0.1	0.1–0.2	37/46 (80.4)	7.2 ± 9.5 (0.1–30.0)	1.9	0.3–9.3
18	5/39 (12.8)	0.2 ± 0.3 (0.1–0.7)	0.1	0.1–0.2	0/39 (0.0)	0.0	0.0	0.0–0.0
19	1/42 (2.4)	0.1^a^	0.1	0.1–0.1	0/42 (0.0)	0.0	0.0	0.0–0.0
20	15/43 (34.9)	0.2 ± 0.1 (0.1–0.6)	0.1	0.1–0.2	15/43 (34.9)	0.3 ± 0.3 (0.1–1.0)	0.2	0.1–0.3
21	10/42 (23.8)	0.4 ± 0.4 (0.1–1.2)	0.3	0.2–0.7	2/42 (4.8)	0.2 ± 0.1 (0.1–0.2)	0.2	0.1–0.2
22	0/49 (0.0)	0.0	0.0	0.0–0.0	3/49 (6.1)	0.2 ± 0.2 (0.1–0.4)	0.1	0.1–0.4
23	1/45 (2.2)	0.1^a^	0.1	0.1–0.1	0/45 (0.0)	0.0	0.0	0.0–0.0
24	10/43 (23.3)	0.2 ± 0.2 (0.1–0.6)	0.2	0.1–0.2	43/43 (100.0)	58.0 ± 67.7 (1.1–292.4)	35.0	15.6–80.3
25	4/38 (10.5)	0.1 ± 0.0 (0.1–0.1)	0.1	0.1–0.1	6/38 (15.8)	0.2 ± 0.1 (0.1–0.2)	0.2	0.1–0.2
26	0/49 (0.0)	0.0	0.0	0.0–0.0	0/49 (0.0)	0.0	0.0	0.0–0.0
27	6/48 (12.5)	0.3 ± 0.2 (0.1–0.7)	0.3	0.1–0.4	2/48 (4.2)	1.6 ± 2.1 (0.1–3.1)	1.6	0.1–3.1
Total	89/667 (13.4)	0.3 ± 0.4 (0.1–2.5)	0.1	0.1–0.3	179/667 (26.8)	25.4 ± 46.2 (0.1–292.4)	7.4	0.3–26.4

^a^Standard deviation and range are not given as only one sample was positive

Co-infections with strongyles and *D. viviparus* were present in 26.7% (4/14) of DS1 herds. Infections with strongyles and *F. hepatica* were detected in 50.0% (7/14) of DS1 herds, however, in one of these herds (herd no. 4), no co-infected individual was among the sampled cows. Furthermore, strongyle and *F. hepatica* co-infections were detected in 86.7% (13/15) of DS2 herds. In addition, 21.4% (3/14) of DS1 and 53.3% (8/15) of DS2 herds were co-infected with strongyles, *F. hepatica* and rumen flukes. All herds affected by rumen flukes were also affected by *F. hepatica*, with the exception of herd no. 22, which was only co-infected with strongyles.

### Frequency of helminth taxa in individual faecal samples of the two datasets

**Fig. 2:**
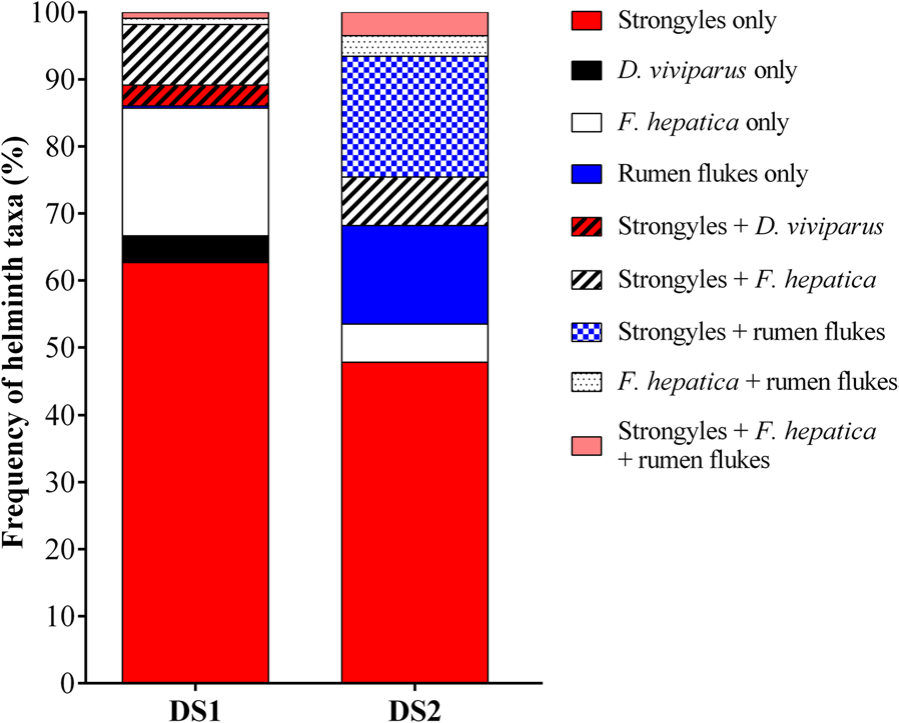
Frequency of helminth mono- and co-infections among positive individual faecal samples in the datasets. DS1: dataset 1; DS2: dataset 2

In DS1, where cows were sampled twice (July and September 2015), patent helminth infections were detected in 36.2% (552/1524) of individual faecal samples. Strongyle eggs were the most frequently detected helminth egg type with 27.4% (418/1524) positive faecal samples. *Dictyocaulus viviparus* larvae, *F. hepatica* eggs and rumen fluke eggs were detected in 2.6% (39/1524), 10.8% (165/1524) and 0.8% (12/1524) of samples, respectively. In total, 31.2% (475/1524) of faecal samples were classified as mono- and 5.0% (77/1524) as co-infected (cf. Table 2). The distribution of helminth taxa considering mono- and co-infections among the 552 positive faecal samples is presented in Fig. 2.

In DS2, a proportion of 46.6% (311/667) of cows were mono- and 21.7% (145/667) of cows were co-infected. More detailed data on helminth taxa in mono- and co-infections among the 456 infected cows is given in Fig. 2.

### Correlations between helminth taxa and differences between mono- and co-infected cows

In DS1, the correlations between strongyle EPG with *D. viviparus* LPG and *F. hepatica* EPG were 0.11 (Pearson correlation, *P* ≤ 0.0001, *df* = 1522) and 0.08 (Pearson correlation, *P* = 0.002, *df* = 1522), respectively. In addition, a significant positive correlation of 0.16 (Pearson correlation*, P* ≤ 0.0001, *df* = 1522) was observed between *F. hepatica* and rumen fluke EPG. The correlations between strongyle EPG with rumen fluke EPG, and between *D. viviparus* LPG with *F. hepatica* and rumen fluke EPG were not significant. No statistically significant correlations were observed in DS2.

The comparison of egg/larvae excretion intensity between mono- and coinfected cows of the two datasets revealed a significantly higher mean *F. hepatica* EPG in DS1 samples presenting co-infections (Mann–Whitney test, *U* = 2774, *P* = 0.013). No further statistical significance was observed. Detailed data are presented in Table 5.

**Table 5:** Results of the Mann–Whitney test on differences in the mean rank of EPG/LPG between faecal samples presenting mono- and co-infections in dataset 1 (DS1) and dataset 2 (DS2) with corresponding *P*-values (significant *P*-values printed in bold). In addition, mean EPG/LPG values with corresponding standard deviation (SD), range (minimum and maximum), median and interquartile range (25th to 75th percentile) in faecal samples presenting mono- and co-infections are listed

	Mono-infected				Co-infected				
	No. of records	Mean EPG/LPG ± SD (range)	Median	Interquartile range	No. of records	Mean EPG/LPG ± SD (range)	Median	Interquartile range	*P*-value
Dataset 1 (DS1)									
Strongyles	346	39.7 ± 23.7 (25.0–150.0)	25.0	25.0–50.0	72	47.5 ± 34.6 (25.0–225.0)	25.0	25.0–50.0	0.072
* D. viviparus*	22	0.2 ± 0.3 (0.03–1.2)	0.05	0.03–0.2	17	0.03 ± 0.03 (0.03–1.1)	0.1	0.03–0.5	0.281
* F. hepatica*	105	0.5 ± 0.8 (0.1–7.1)	0.2	0.1–0.7	60	0.9 ± 1.3 (0.1–8.9)	0.4	0.2–1.0	**0.013**
Dataset 2 (DS2)									
Strongyles	218	6.8 ± 9.9 (2.0–84.0)	4.0	2.0–6.0	131	6.4 ± 7.3 (2.0–54.0)	4.0	2.0–8.0	0.246
* F. hepatica*	26	0.4 ± 0.5 (0.1–2.5)	0.1	0.1–0.5	63	0.3 ± 0.4 (0.1–1.9)	0.2	0.1–0.3	0.9082
Rumen flukes	67	26.8 ± 46.2 (0.1–206.8)	6.4	0.3–24.7	112	24.5 ± 46.4 (0.1–292.4)	7.5	0.0–0.1	0.9911

### Selection and validation of linear mixed models

For DS1, the mean of three test-day milk production records after the faecal examination resulted in the smallest AIC. Furthermore, as most of the DS1 herds suspended test-day recording in June or July 2015, the number of cows with milk production data was highest when using the mean of the three test-day milk production records after the faecal examination. Therefore, this value was used in DS1 cows for milk yield, milk protein content and milk fat content in July 2015 as well as in September 2015.

For DS2, where the cows were sampled over a wide range of months (May 2021 to February 2022) due to the given study design, AIC value was smallest when using the mean of both test-day milk production records within 30 days before and 30 days after the faecal examination, and thus used for milk yield, milk protein content and milk fat content in DS2 cows.

The residuals from model 1 and 2 and the random variances from model 1 were approximately normally distributed according to the results from quantile–quantile plots, but did not follow a Gaussian distribution according to the results from the Shapiro–Wilk test.

### Impact of infection status on milk yield and milk quality

The overall *F*-test of linear mixed models 1 and 2 revealed that the infection status (non-infected *vs*. mono-infected *vs*. co-infected) had no significant effect on milk yield or milk protein and fat percentage, neither for DS1 nor for the two DS2 classifications. Least-squares means for milk yield, protein content and fat content in non-infected, mono- and co-infected cows in DS1 and DS2 are presented in Table 6. Further detailed results of linear mixed model 1 and 2 are given in Additional file 1: Table S1–Table S3.

**Table 6:** Least-squares means with corresponding standard error (± SE) and *P*-values (results from overall F-tests) for milk production parameters from linear mixed model 1 and 2

Infection status^1^	MY-DS1	P%-DS1	F%-DS1	MY-DS2	P%-DS2	F%-DS2
DS1 & DS2 (classification 1)						
Non-infected	21.44 ± 0.54^a^	3.26 ± 0.06^a^	4.21 ± 0.08^a^	26.43 ± 0.80^a^	3.46 ± 0.04^a^	4.03 ± 0.07^a^
Mono-infected	21.42 ± 0.57^a^	3.24 ± 0.06^a^	4.18 ± 0.09^a^	25.76 ± 0.72^a^	3.45 ± 0.03^a^	4.02 ± 0.06^a^
Co-infected	21.25 ± 0.85^a^	3.30 ± 0.09^a^	4.35 ± 0.13^a^	25.59 ± 0.86^a^	3.47 ± 0.04^a^	4.07 ± 0.08^a^
*P*-value	0.9665	0.7503	0.2563	0.4501	0.8110	0.7187
DS2 (classification 2)						
Non-infected	–	–	–	26.46 ± 0.81^a^	3.47 ± 0.04^a^	4.04 ± 0.07^a^
Mono-infection status 1	–	–	–	25.86 ± 0.77^a^	3.45 ± 0.04^a^	4.05 ± 0.07^a^
Mono-infection status 2	–	–	–	25.23 ± 1.39^a^	3.46 ± 0.07^a^	3.95 ± 0.12^a^
Mono-infection status 3	–	–	–	25.60 ± 1.17^a^	3.44 ± 0.06^a^	4.08 ± 0.10^a^
Co-infection status 1	–	–	–	25.59 ± 1.26^a^	3.53 ± 0.06^a^	4.08 ± 0.11^a^
Co-infections status 2	–	–	–	25.95 ± 1.10^a^	3.44 ± 0.05^a^	4.05 ± 0.10^a^
Co-infection status 3	–	–	–	24.00 ± 1.79^a^	3.41 ± 0.09^a^	3.89 ± 0.16^a^
Co-infection status 4	–	–	–	24.81 ± 1.74^a^	3.42 ± 0.08^a^	4.10 ± 0.15^a^
*P*-value	–	–	–	0.8521	0.9182	0.8189

SE: standard error; DS1: dataset 1; DS2: dataset 2; MY: milk yield (in kg); P%: protein percentage; F%: fat percentage

^1^The infection status (classification 1 and classification 2) used in linear mixed model 1 and 2 is described in Table 2

^a^Indicates no statistical significant differences (*P* > 0.05) between fixed effect classes within each column

## Discussion

We present the first study analysing the impact of patent helminth mono-infections versus co-infections on milk yield and milk quality in dairy cows. Two datasets of dairy herds contributing to different research projects were used in our analyses. Co-infections were detected in 81.5% and 93.3% of DS1 and DS2 herds, respectively. Two recently published seroprevalence studies conducted in northern and southern Germany reported a co-exposure to *O. ostertagi* and *F. hepatica* in 12.8 to 22.4% of herds based on BTM antibody levels [5, 15]. In our study, patent co-infections between strongyles and *F. hepatica* were present in 57.1% of herds in DS1 and 86.7% of herds in DS2. The higher co-infection rate in our datasets can be explained by a preselection of dairy herds. The DS1 herds were selected based on the criteria that all cows had pasture access since April/May 2015 and were not treated with anthelmintics in the sampling year. For DS2 herds, proven patent *F. hepatica* infections or increased BTM *F. hepatica* antibody levels were a requirement for selection. In addition, anthelmintic treatment was only conducted in four DS2 farms in dry cows and therefore not considered in the linear mixed model analyses. As a further consequence of the preselection criteria for DS2, 60% (9/15) of DS2 herds were infected with rumen flukes, which use the same intermediate host, and eight of these herds were co-infected with *F. hepatica*. Interestingly, herd no. 1 was positive for rumen flukes in 2021, but rumen fluke eggs were not detected in 2015. Vice versa, herd no. 2 switched from a positive infection status for rumen flukes on 2015 to a negative status in 2022, although no anthelmintic treatment was applied. Similar to our findings on herd level, Jones et al. [9] found 46.0% of cattle herds in the UK to be co-infected with *F. hepatica* and rumen flukes in faecal examinations.

The use of two different datasets pre-selected for different research projects also entails that exposure to helminth infections in DS1 and DS2 may have differed due to geographical location of herds as well as year and season of faecal examination. While the majority of DS2 herds were located in northern Germany with wetter climatic conditions (i.e., high rainfall), five of the 14 DS1 herds were located in the western part and in the middle of Germany with drier weather conditions, especially in 2015. The DS1 cows were examined in July with a high probability for strongyle egg excretion, and in September, a month with a relatively high probability to detect patent *D. viviparus* and *F. hepatica* infections. In contrast, the DS2 cows were examined once during the period from May 2021 to February 2022. Here, a cow sampled during the winter period could be classified as negative due to egg or larvae excretion below the detection limit of the coproscopical method, although potentially infected with high numbers of hypobiotic *O. ostertagi* or *D. viviparus* larvae. Even though hypobiotic *O. ostertagi* and *D. viviparus* larvae are often harboured by clinically asymptomatic carrier animals [21, 22], subclinical effects may have influenced milk production parameters and thus to some extent our association analysis, as only patent (co)-infections were considered. According to our knowledge, the effect of infections with hypobiotic larvae on milk production in dairy cows is still unclear and remains to be investigated in future studies. In sheep experimentally infected with *Haemonchus contortus*, a significantly lower weight gain was observed in animals infected with hypobiotic larvae compared to the uninfected control group [23]. Moreover, a recent study found a significant negative effect of early (migrating) *F. hepatica* infection on weight gain in young cattle [24].

In addition, the diagnostic method to detect strongylid eggs and its sensitivity differed between DS1 and DS2. In the latter, the FLOTAC double method with an analytical sensitivity of 2 EPG was used compared to the modified McMaster method with an analytical sensitivity of 25 EPG in DS1, explaining the higher prevalence of 52.3% for strongyles in DS2 compared to 27.4% in DS1 and discrepant prevalences within the same herds (i.e., herd no. 1 and 2). *Fasciola hepatica* and rumen fluke eggs were determined with the sedimentation technique on 10 g faeces. Using this amount, the sensitivity of the sedimentation technique was shown to be similar to the alternative Flukefinder® method [18, 25].

The cows in DS1 were classified in non-infected, mono-infected and co-infected independent from the detected helminth infection, as the prevalence for *D. viviparus* and rumen flukes was only 2.6% and 0.8% and thus too low to include *D. viviparus* and rumen fluke mono-infections and co-infections with other helminth taxa as fixed effects in model 1. Thus, co-infections in DS1 mainly represent co-infections between strongyles and *F. hepatica*. A more detailed classification (classification 2, cf. Table 2) modelling strongyle, *F. hepatica* and rumen fluke infections as mono- and co-infections was possible in DS2 with prevalences of 52.3%, 13.3% and 26.8%, respectively. Due to the differences in faecal examination methods and in rumen fluke prevalence between DS1 and DS2, we applied separate linear mixed model analyses for the two datasets to investigate the effect of infection status on milk production parameters.

A decline in milk production parameters in response to helminth infections was estimated in several studies [1]. For infections with *D. viviparus*, milk yield was reduced by up to 1.68 kg/cow/day in positive herds compared to negative herds as determined by herd antibody levels [26], and in patently infected compared to non-infected cows as assessed by the Baermann technique [16]. Moreover, significant negative associations were estimated between GIN infections detected by *O. ostertagi* BTM antibody levels with milk yield and milk quality [27, 28]. For *F. hepatica* infections, May et al. [7] reported a loss of 1.62 kg milk/cow/day and a decrease of 0.06 kg milk protein and fat yield in BTM ELISA positive compared to negative dairy herds. However, the current published studies strongly focussed on a single helminth infection by neglecting co-infections with other helminth species since co-infections were mostly not examined. Other studies investigated co-infections but were not able to model the impact of co-infections between two or more helminth species on milk production parameters since the frequency of one helminth species in the copromicroscopical examination was too low [14].

In our study, we had a sufficiently high proportion of patently co-infected cows to estimate differences in milk production traits between non-infected, mono- and co-infected cows. We hypothesised an additive detrimental impact on milk yield and milk quality in cows being patently co-infected with two or more helminth species compared to patently mono-infected cows. However, we estimated no additive or synergistic effect of helminth co-infections on milk yield and milk quality. Our findings indicate that farmers do not need to pay special attention to their cows if co-infections have been detected on the farm. This is consistent with the finding by Charlier et al. [6], who observed no additive or synergistic effect of *O. ostertagi* and *F. hepatica* co-exposure on milk yield compared to herds exposed to only one of the helminth species when the interaction term of *O. ostertagi* and *F. hepatica* BTM antibody levels was included in the statistical model. While Charlier et al. [6] associated BTM ELISA results with the mean milk yield per herd per month or year, our model analyses based on individual cow test-day data and proven patent infections, providing a more accurate association between the actual infection status and milk production parameters compared to studies based on herd level. However, association analyses modelling the impact of helminth (co-)infections on milk production parameters present a challenge due to discrepancies in the prepatent period of different helminth taxa. For strongyles and *D. viviparus*, the prepatent period is about three weeks [29, 30], while it is 8–12 weeks and 12–14 weeks for *F. hepatica* and rumen flukes [31, 32], respectively. However, as at the usual pasture contamination in Central Europe (sub-)clinical effects in dairy cows are only expected after several weeks of parasite accumulation, we only considered a time period for milk production test-day recording of 30–40 days before faecal examination in the model selection process. This minimised the risk for failing potential prepatent infection effects and increased the number of milk production test-day records per cow before faecal examination to improve model quality. We tested different time periods between faecal examination and milk production test-day records, and means of milk production records compared to single test-day records, prior to the selection of the final model for DS1 and DS2. Finally, we used the mean of one to three monthly milk production test-day records before and/or after the faecal examination, because AIC values were smallest for the chosen mean instead of linking only one monthly single test-day for all cows to one faecal examination. However, AIC values were very similar between different means of several milk production test-day records or using only a single milk production test-day record in DS1 and DS2. Moreover, results of the overall F-test and differences of LSM were almost the same when using means of test-day records or single test-days (result not shown). The residuals and random variances from linear mixed model 1 and 2 were only approximately normally distributed, which may limit the power of our model analyses. However, Schielzeth et al. [33] showed that linear mixed models are robust for such minor violations in assumptions of normality.

When investigating helminth effects on milk production, the question arises whether the effect of different taxa on milk production is short-term or long-term. For *F. hepatica*, we hypothesised a possible long-term effect on milk production parameters since cattle are usually chronically infected, while the effect of strongyle and *D. viviparus* infections was assumed to be more short-term. Hence, we also tested such possible effects during the model selection process (not shown in the manuscript): We tested the association between *F. hepatica* infections and the mean of milk production parameters for six months after faecal examination in DS1 and DS2 as a possible long-term effect. However, this association was not statistically significant. Furthermore, we ran models within *F. hepatica* positive herds analysing the association between *F. hepatica* infection status (infected vs. non-infected) and milk production during the whole lactation period. To test a possible short-term effect, we ran models between *F. hepatica* infection status and the milk production test-day after faecal examination. The results of these analyses (data not included in the manuscript) showed a significant short-term association between patent *F. hepatica* infections and milk production test-day after faecal examination, but no long-term effect when considering the whole lactation period in within-herd analyses. Since we modelled co-infections (e.g., with *F. hepatica* and strongyles) in our study, it was not possible to model short-term or long-term effects simultaneously.

Overall, co-infections were present in only 5.0% and 21.7% of individual faecal samples in DS1 and DS2, respectively. On the one hand, it is possible that the co-infection rates in our datasets provided from two field studies are too small, especially in DS1, to derive a statistical association between infection status and milk production parameters. On the other hand, when ignoring co-infections in the two datasets and modelling the infection status for each helminth taxon separately, we additionally estimated no or only minor effects of helminth infections on milk production parameters (results previously partly shown for DS1 [4, 14, 16]). For rumen flukes, Maltrait et al. [34] proposed a cut-off of 200 EPG to detect highly infected animals (> 200 flukes). In our study, EPGs above 200 were present only in 0.5% (3/667) of DS2 cows, indicating a generally low rumen fluke infection intensity in examined dairy cows. Moreover, for rumen flukes, the pathogenicity and clinical relevance in cattle is still unclear. Also, the egg and larvae counts for strongyles, *D. viviparus* and *F. hepatica* were quite low with a maximum of 225.0 EPG, 1.2 LPG and 8.9 EPG in 2015, and a maximum of 84.0 EPG, 0.0 LPG and 2.5 EPG in 2021/2022. In dairy cows, low EPGs and LPGs can be expected due to adaptive immunity or improved immune response to reinfections. Burden et al. [35] observed no differences in EPGs in calves mono-infected with either *F. hepatica* or *O. ostertagi*, or co-infected with both helminth species. Similar results were observed in the present study, as significant differences in the mean egg or larvae counts between mono- and co-infected cows were only found for *F. hepatica* in DS1. Hence, we hypothesise that low worm burdens in the cows contribute to the non-significant relationship between infection status and milk production parameters.

The correlations between EPG and LPG values were mainly close to zero in both datasets. We only observed a significant positive correlation of 0.11 between strongyle EPG with *D. viviparus* LPG, and of 0.16 between *F. hepatica* EPG and rumen fluke EPG in DS1. In contrast, Jones et al. [9] reported a significant negative correlation of − 0.35 between EPG of *F. hepatica* and the rumen fluke species *Calicophoron daubneyi*. The negative correlation was explained by treatment of cows against *F. hepatica* with anthelmintics being not active against rumen flukes. An explanation for the positive correlation between strongyle egg and *D. viviparus* larvae shedding might be a more similar immune response of cattle against *D. viviparus* and strongyles (mixed Th1/Th2 response) [36], instead of the classical Th2 dominated immune response against *F. hepatica*. The positive correlation of 0.16 for *F. hepatica* and rumen flukes in DS1 might be explained by the fact that both trematodes share the same intermediate host, implying that *F. hepatica* and rumen flukes are likely to be ingested simultaneously during grazing. Further studies are needed to investigate the underlying mechanisms in management and cow physiology explaining positive or negative correlations in egg and larvae counts between different helminth taxa.

## Conclusions

In the presented study, we used two different datasets to analyse helminth co-infections and the association between infection status (non-infected *vs.* mono-infected *vs.* co-infected) and milk production parameters in dairy cows. Co-infections with more than one helminth taxa were present in 81.5% of herds examined in 2015 and in 93.3% of herds examined in 2021/2022. The percentage of faecal samples presenting co-infections with more than one helminth taxa ranged from 5.0 to 21.7% in the two datasets. Our results indicate that co-infections with two or more helminth taxa have no additive and no synergistic impact on dairy cow milk production parameters compared to mono-infections or no infection. Although we expected additive or synergistic effects of helminth co-infections, the risk for increased economic losses due to co-infections appears to be low. This finding might be a result of low EPGs or low worm burdens in dairy cows. Future studies in more severely infected herds are therefore desirable to investigate the impact of helminth co-infections on milk production parameters in dairy cows.

## Supplementary Information

Additional file 1.

Additional file 2.

## Data Availability

Data is provided within the manuscript or supplementary information files.

## Abbreviations

BTM: Bulk tank milk
DIM: Days in milk
ELISA: Enzyme immunosorbent assay
EPG: Eggs per gram faeces
F%-DS1: Fat percentage in dataset 1
F%-DS2: Fat percentage in dataset 2
LPG: Larvae per gram faeces
LSM: Least-squares means
MY-DS1: Milk yield (in kg) in dataset 1
MY-DS2: Milk yield (in kg) in dataset 2
P%-DS1: Protein percentage in dataset 1
P%-DS2: Protein percentage in dataset 2
