# Supplementary material for: Helminth co-infections have no additive detrimental impact on milk yield and milk quality compared to mono-infections in German dairy cows
Source: Parasit Vectors. 2024 Sep 19;17:398. doi: 10.1186/s13071-024-06470-8 (PMC11414050; doi:10.1186/s13071-024-06470-8)
Supplement: Supplementary file 1 — Additional file 1. [file 13071_2024_6470_MOESM1_ESM.docx]

Table S1. Number of observations within effect classes (N), least-squares means with corresponding standard error (± SE) and *P*-values (results from overall F-tests) for milk production parameters (milk yield, milk protein and fat percentage) from linear mixed model 1 applied on dataset 1 (DS1).

| **Fixed effects** | **Effect class** | **N** | **MY-DS1** | **P%-DS1** | **F%-DS1** |
| --- | --- | --- | --- | --- | --- |
| Infection status | Non-infected | 972 | 21.44 ± 0.54 | 3.26 ± 0.06 | 4.21 ± 0.08 |
|  | Mono-infected | 475 | 21.42 ± 0.57 | 3.24 ± 0.06 | 4.18 ± 0.09 |
|  | Co-infected | 77 | 21.25 ± 0.85 | 3.30 ± 0.09 | 4.35 ± 0.13 |
|  | *P*-value |  | 0.9665 | 0.7148 | 0.2563 |
| Herd (no.) | 1 | 114 | 18.42 ± 0.93 | 3.33 ± 0.08 | 4.18 ± 0.15 |
|  | 2 | 47 | 17.86 ± 1.00 | 3.43 ± 0.11 | 4.78 ± 0.15 |
|  | 3 | 95 | 21.75 ± 0.78 | 3.31 ± 0.08 | 4.44 ± 0.12 |
|  | 4 | 117 | 24.51 ± 0.75 | 3.26 ± 0.08 | 3.91 ± 0.12 |
|  | 5 | 330 | 21.66 ± 0.83 | 3.28 ± 0.07 | 4.11 ± 0.13 |
|  | 6 | 94 | 21.65 ± 0.84 | 3.34 ± 0.09 | 4.19 ± 0.13 |
|  | 7 | 119 | 24.75 ± 0.78 | 3.20 ± 0.08 | 4.39 ± 0.12 |
|  | 8 | 68 | 20.28 ± 0.93 | 3.38 ± 0.10 | 4.46 ± 0.14 |
|  | 9 | 127 | 18.08 ± 0.76 | 3.85 ± 0.08 | 3.66 ± 0.12 |
|  | 10 | 85 | 22.08 ± 0.83 | 3.22 ± 0.09 | 4.17 ± 0.13 |
|  | 11 | 49 | 21.54 ± 0.99 | 3.32 ± 0.11 | 4.51 ± 0.16 |
|  | 12 | 49 | 22.80 ± 0.97 | 3.28 ± 0.11 | 4.12 ± 0.15 |
|  | 13 | 129 | 23.53 ± 0.75 | 3.39 ± 0.08 | 4.57 ± 0.12 |
|  | 14 | 101 | 20.84 ± 0.92 | 3.14 ± 0.09 | 3.95 ± 0.15 |
|  | *P*-value |  | <0.0001 | <0.0001 | <0.0001 |
| Genetic line | DSN | 532 | 20.55 ± 0.79 | - | 4.22 ± 0.12 |
|  | GHm | 823 | 22.28 ± 0.58 | - | 4.10 ± 0.09 |
|  | GHp | 76 | 21.42 ± 0.83 | - | 4.17 ± 0.13 |
|  | GHnz | 93 | 21.23 ± 0.83 | - | 4.49 ± 0.13 |
|  | *P*-value |  | 0.0350 | - | 0.0016 |
| Parity number | 1 | 409 | 18.87 ± 0.62 | - | - |
|  | 2 | 409 | 21.16 ± 0.60 | - | - |
|  | 3 | 270 | 22.72 ± 0.65 | - | - |
|  | 4 | 194 | 22.13 ± 0.69 | - | - |
|  | >=5 | 242 | 21.97 ± 0.68 | - | - |
|  | *P*-value |  | <0.0001 | - | - |
| Days in milk | <=10 | 92 | 26.51 ± 1.11 | 2.98 ± 0.11 | 4.08 ± 0.17 |
|  | 11 to 50 | 252 | 25.02 ± 0.89 | 3.13 ± 0.09 | 4.19 ± 0.14 |
|  | 51 to 100 | 215 | 21.85 ± 0.70 | 3.32 ± 0.07 | 4.28 ± 0.11 |
|  | 101 to 200 | 385 | 17.95 ± 0.58 | 3.57 ± 0.06 | 4.50 ± 0.09 |
|  | >=201 | 580 | 15.52 ± 0.56 | 3.33 ± 0.06 | 4.17 ± 0.09 |
|  | *P-*value |  | <0.0001 | <0.0001 | <0.0001 |
| Season | Summer | 732 | 21.91 ± 0.54 | - | 4.09 ± 0.08 |
|  | Autumn | 792 | 20.83 ± 0.65 | - | 4.40 ± 0.10 |
|  | *P-*value |  | 0.0018 | - | <0.0001 |
| Calving time | Jan – Mar 2014 | 7 | 21.59 ± 2.22 | 3.56 ± 0.25 | 4.66 ± 0.35 |
|  | Apr – Jun 2014 | 19 | 20.05 ± 1.48 | 3.14 ± 0.16 | 4.15 ± 0.23 |
|  | Jul – Sep 2014 | 259 | 22.33 ± 0.84 | 2.89 ± 0.08 | 3.86 ± 0.13 |
|  | Oct – Dec 2014 | 314 | 22.36 ± 0.65 | 3.19 ± 0.07 | 4.24 ± 0.11 |
|  | Jan – Mar 2015 | 395 | 21.92 ± 0.55 | 3.29 ± 0.05 | 4.30 ± 0.08 |
|  | Apr – Jun 2015 | 282 | 22.13 ± 0.49 | 3.28 ± 0.05 | 4.19 ± 0.08 |
|  | Jul – Sep 2015 | 248 | 20.18 ± 0.72 | 3.51 ± 0.07 | 4.33 ± 0.11 |
|  | *P*-value |  | 0.0306 | <0.0001 | <0.0001 |

SE, standard error; DS1, dataset 1; MY, milk yield (in kg); P%, protein percentage; F%, fat percentage

**Table S2.** Number of observations within effect classes (N), least-squares means with corresponding standard error (± SE) and *P*-values (results from overall F-tests) for milk production parameters (milk yield, milk protein and fat percentage) from linear mixed model 2 applied on dataset 2 (DS2, infection status according to classification 1).

| **Fixed effects / covariates** | **Effect class** | **N** | **MY-DS2** | **P%-DS2** | **F%-DS2** |
| --- | --- | --- | --- | --- | --- |
| Infection status | Non-infected | 211 | 26.43 ± 0.80 | 3.46 ± 0.04 | 4.03 ± 0.07 |
|  | Mono-infected | 311 | 25.76 ± 0.72 | 3.45 ± 0.03 | 4.02 ± 0.06 |
|  | Co-infected | 145 | 25.59 ± 0.86 | 3.47 ± 0.04 | 4.07 ± 0.08 |
|  | *P*-value |  | 0.4501 | 0.8110 | 0.7187 |
| Herd (no.) | 1 | 49 | 13.74 ± 1.49 | 3.93 ± 0.07 | 4.78 ± 0.13 |
|  | 2 | 33 | 21.72 ± 2.13 | 3.21 ± 0.10 | 4.20 ± 0.19 |
|  | 15 | 49 | 28.56 ± 1.01 | 3.53 ± 0.05 | 4.15 ± 0.09 |
|  | 16 | 52 | 25.31 ± 0.97 | 3.41 ± 0.05 | 3.89 ± 0.09 |
|  | 17 | 46 | 29.60 ± 1.09 | 3.55 ± 0.05 | 3.99 ± 0.10 |
|  | 18 | 39 | 24.04 ± 1.16 | 3.44 ± 0.06 | 3.98 ± 0.10 |
|  | 19 | 42 | 33.50 ± 1.02 | 3.43 ± 0.05 | 3.96 ± 0.09 |
|  | 20 | 43 | 17.30 ± 1.12 | 3.62 ± 0.05 | 4.31 ± 0.10 |
|  | 21 | 42 | 32.96 ± 1.00 | 3.31 ± 0.05 | 3.88 ± 0.09 |
|  | 22 | 49 | 25.00 ± 1.25 | 3.47 ± 0.06 | 3.99 ± 0.11 |
|  | 23 | 45 | 30.75 ± 0.99 | 3.29 ± 0.05 | 3.78 ± 0.09 |
|  | 24 | 43 | 26.80 ± 1.16 | 3.40 ± 0.06 | 3.72 ± 0.10 |
|  | 25 | 38 | 25.82 ± 1.98 | 3.03 ± 0.10 | 3.58 ± 0.18 |
|  | 26 | 49 | 27.03 ± 1.24 | 3.68 ± 0.06 | 4.20 ± 0.11 |
|  | 27 | 48 | 26.77 ± 1.02 | 3.63 ± 0.05 | 4.19 ± 0.09 |
|  | *P*-value |  | <0.0001 | <0.0001 | <0.0001 |
| Parity number | 1 | 190 | 23.68 ± 0.78 | 3.43 ± 0.04 | 4.00 ± 0.07 |
|  | 2 | 168 | 25.43 ± 0.80 | 3.53 ± 0.04 | 4.15 ± 0.07 |
|  | 3 | 127 | 26.61 ± 0.86 | 3.47 ± 0.04 | 4.03 ± 0.08 |
|  | 4 | 69 | 26.43 ± 0.94 | 3.50 ± 0.05 | 4.05 ± 0.08 |
|  | >4 | 113 | 27.49 ± 0.87 | 3.38 ± 0.04 | 3.97 ± 0.08 |
|  | *P*-value |  | <0.0001 | 0.0002 | 0.0549 |
| Calving time | Jan – Mar 2020 | 8 | 30.95 ± 3.87 | 3.20 ± 0.19 | 3.62 ± 0.34 |
|  | Apr – Jun 2020 | 25 | 23.76 ± 2.76 | 3.43 ± 0.13 | 3.61 ± 0.25 |
|  | Jul – Sep 2020 | 52 | 22.53 ± 1.91 | 3.55 ± 0.09 | 4.04 ± 0.17 |
|  | Oct – Dec 2020 | 123 | 23.84 ± 1.05 | 3.50 ± 0.05 | 4.11 ± 0.09 |
|  | Jan – Mar 2021 | 196 | 25.99 ± 0.48 | 3.51 ± 0.02 | 4.11 ± 0.04 |
|  | Apr – Jun 2021 | 113 | 26.60 ± 0.82 | 3.48 ± 0.04 | 4.06 ± 0.07 |
|  | Jul – Sep 2021 | 112 | 27.30 ± 1.50 | 3.45 ± 0.07 | 4.29 ± 0.13 |
|  | Oct – Dec 2021 | 38 | 26.44 ± 2.30 | 3.59 ± 0.11 | 4.49 ± 0.20 |
|  | *P*-value |  | 0.0006 | 0.0151 | 0.0064 |
| Mean days in milk | *P*-value |  | 0.0090 | 0.0001 | 0.0072 |

SE, standard error; DS2, dataset 2; MY, milk yield (in kg); P%, protein percentage; F%, fat percentage

Table S3. Number of observations within effect classes (N), least-squares means with corresponding standard error (± SE) and *P*-values (results from overall F-tests) for milk production parameters (milk yield, milk protein and fat percentage) from linear mixed model 2 applied on dataset 2 (DS2, infection status according to classification 2).

| **Fixed effects / covariates** | **Effect class** | **N** | **MY-DS2** | **P%-DS2** | **F%-DS2** |
| --- | --- | --- | --- | --- | --- |
| Infection status | Non-infected | 211 | 26.46 ± 0.81 | 3.47 ± 0.04 | 4.04 ± 0.07 |
|  | Mono-infection status 1 | 218 | 25.86 ± 0.77 | 3.45 ± 0.04 | 4.05 ± 0.07 |
|  | Mono-infection status 2 | 26 | 25.23 ± 1.39 | 3.46 ± 0.07 | 3.95 ± 0.12 |
|  | Mono-infection status 3 | 67 | 25.60 ± 1.17 | 3.44 ± 0.06 | 4.08 ± 0.10 |
|  | Co-infection status 1 | 33 | 25.59 ± 1.26 | 3.53 ± 0.06 | 4.08 ± 0.11 |
|  | Co-infections status 2 | 82 | 25.95 ± 1.10 | 3.44 ± 0.05 | 4.05 ± 0.10 |
|  | Co-infection status 3 | 14 | 24.00 ± 1.79 | 3.41 ± 0.09 | 3.89 ± 0.16 |
|  | Co-infection status 4 | 16 | 24.81 ± 1.74 | 3.42 ± 0.08 | 4.10 ± 0.15 |
|  | *P*-value |  | 0.8521 | 0.9182 | 0.8189 |
| Herd (no.) | 1 | 49 | 13.16 ± 1.53 | 3.93 ± 0.07 | 4.76 ± 0.14 |
|  | 2 | 33 | 21.09 ± 2.20 | 3.20 ± 0.11 | 4.15 ± 0.19 |
|  | 15 | 49 | 28.09 ± 1.23 | 3.51 ± 0.05 | 4.11 ± 0.10 |
|  | 16 | 52 | 24.81 ± 1.08 | 3.43 ± 0.05 | 3.90 ± 0.10 |
|  | 17 | 46 | 29.28 ± 1.19 | 3.55 ± 0.06 | 4.02 ± 0.11 |
|  | 18 | 39 | 23.50 ± 1.28 | 3.43 ± 0.06 | 3.93 ± 0.11 |
|  | 19 | 42 | 32.99 ± 1.15 | 3.41 ± 0.06 | 3.91 ± 0.10 |
|  | 20 | 43 | 16.93 ± 1.14 | 3.61 ± 0.06 | 4.28 ± 0.10 |
|  | 21 | 42 | 32.56 ± 1.10 | 3.29 ± 0.05 | 3.86 ± 0.10 |
|  | 22 | 49 | 24.41 ± 1.35 | 3.46 ± 0.07 | 3.94 ± 0.12 |
|  | 23 | 45 | 30.25 ± 1.13 | 3.28 ± 0.05 | 3.74 ± 0.10 |
|  | 24 | 43 | 26.47 ± 1.25 | 3.41 ± 0.06 | 3.74 ± 0.11 |
|  | 25 | 38 | 25.23 ± 2.03 | 3.02 ± 0.10 | 3.54 ± 0.18 |
|  | 26 | 49 | 26.45 ± 1.36 | 3.66 ± 0.07 | 4.15 ± 0.12 |
|  | 27 | 48 | 26.35 ± 1.12 | 3.62 ± 0.05 | 4.15 ± 0.10 |
|  | *P*-value |  | <0.0001 | <0.0001 | <0.0001 |
| Parity number | 1 | 190 | 23.15 ± 0.87 | 3.42 ± 0.04 | 3.97 ± 0.08 |
|  | 2 | 168 | 24.93 ± 0.89 | 3.52 ± 0.04 | 4.12 ± 0.08 |
|  | 3 | 127 | 26.17 ± 0.91 | 3.47 ± 0.04 | 4.01 ± 0.08 |
|  | 4 | 69 | 25.97 ± 1.00 | 3.49 ± 0.05 | 4.02 ± 0.09 |
|  | >4 | 113 | 26.97 ± 0.94 | 3.37 ± 0.05 | 3.94 ± 0.08 |
|  | *P*-value |  | <0.0001 | 0.0003 | 0.0567 |
| Calving time | Jan – Mar 2020 | 8 | 30.39 ± 3.92 | 3.18 ± 0.19 | 3.60 ± 0.35 |
|  | Apr – Jun 2020 | 25 | 23.22 ± 2.80 | 3.42 ± 0.13 | 3.57 ± 0.25 |
|  | Jul – Sep 2020 | 52 | 21.91 ± 1.96 | 3.54 ± 0.09 | 4.01 ± 0.17 |
|  | Oct – Dec 2020 | 123 | 23.32 ± 1.07 | 3.49 ± 0.05 | 4.08 ± 0.10 |
|  | Jan – Mar 2021 | 196 | 25.46 ± 0.60 | 3.50 ± 0.03 | 4.08 ± 0.05 |
|  | Apr – Jun 2021 | 113 | 26.18 ± 0.88 | 3.47 ± 0.04 | 4.04 ± 0.08 |
|  | Jul – Sep 2021 | 112 | 26.96 ± 1.54 | 3.44 ± 0.07 | 4.27 ± 0.14 |
|  | Oct – Dec 2021 | 38 | 26.08 ± 2.33 | 3.58 ± 0.11 | 4.46 ± 0.21 |
|  | *P*-value |  | 0.0005 | 0.0178 | 0.0063 |
| Mean days in milk | *P*-value |  | 0.0114 | 0.0002 | 0.0068 |

SE, standard error; DS2, dataset 2; MY, milk yield (in kg); P%, protein percentage; F%, fat percentage
