# Supplementary material for: Helminth co-infections have no additive detrimental impact on milk yield and milk quality compared to mono-infections in German dairy cows
Source: Parasit Vectors. 2024 Sep 19;17:398. doi: 10.1186/s13071-024-06470-8 (PMC11414050; doi:10.1186/s13071-024-06470-8)
Supplement: Supplementary file 2 — Additional file 2. [file 13071_2024_6470_MOESM2_ESM.docx]

Table S1: In-herd prevalence for strongyles and *D. viviparus* and mean egg or larvae count per gram faeces (EPG/LPG) among all herd samples (positives and negatives) with corresponding standard deviation (SD), range (minimum and maximum), median and interquartile range (25th to 75th percentile) in dataset 1 (DS1) and dataset 2 (DS2).

|  | **Strongyles** | | | | ***D. viviparus*** | | | |
| --- | --- | --- | --- | --- | --- | --- | --- | --- |
| **Herd (no.)** | Positive faecal samples (%) | Mean EPG ± SD (range) | Median | Interquartile range | Positive faecal samples (%) | Mean LPG ± SD (range) | Median | Interquartile range |
| **Dataset 1 (DS1)** | | | | | | | | |
| 1 | 12/81 (14.8) | 3.7 ± 11.6  (0.0 – 75.0) | 0.0 | 0.0 – 0.0 | 0/81 (0.0) | 0.0 | 0.0 | 0.0 – 0.0 |
| 2 | 9/47 (19.2) | 7.4 ± 20.8  (0.0 – 125.0) | 0.0 | 0.0 – 0.0 | 4/47 (8.5) | 0.02 ± 0.07  (0.0 – 0.5) | 0.0 | 0.0 – 0.0 |
| 3 | 17/55 (30.9) | 6.1 ± 13.5  (0.0 – 50.0) | 0.0 | 0.0 – 0.0 | 7/55 (12.7) | 0.004 ± 0.02  (0.0 – 0.13) | 0.0 | 0.0 – 0.0 |
| 4 | 20/66 (30.3) | 10.7 ± 25.1  (0.0 – 150.0) | 0.0 | 0.0 – 0.0 | 0/66 (0.0) | 0.0 | 0.0 | 0.0 – 0.0 |
| 5 | 85/196 (43.4) | 14.2 ± 28.5  (0.0 – 225.0) | 0.0 | 0.0 – 25.0 | 25/196 (12.8) | 0.02 ± 0.13  (0.0 – 1.15) | 0.0 | 0.0 – 0.0 |
| 6 | 27/52 (51.9) | 11.2 ± 19.3  (0.0 – 100.0) | 0.0 | 0.0 – 25.0 | 0/52 (0.0) | 0.0 | 0.0 | 0.0 – 0.0 |
| 7 | 33/68 (48.5) | 12.8 ± 23.2  (0.0 – 125.0) | 0.0 | 0.0 – 25.0 | 0/68 (0.0) | 0.0 | 0.0 | 0.0 – 0.0 |
| 8 | 19/44 (43.2) | 11.8 ± 20.9  (0.0 – 100.0) | 0.0 | 0.0 – 25.0 | 0/44 (0.0) | 0.0 | 0.0 | 0.0 – 0.0 |
| 9 | 42/82 (51.2) | 15.7 ± 22.9  (0.0 – 75.0) | 0.0 | 0.0 – 25.0 | 0/82 (0.0) | 0.0 | 0.0 | 0.0 – 0.0 |
| 10 | 15/48 (31.2) | 6.8 ± 15.1  (0.0 – 75.0) | 0.0 | 0.0 – 0.0 | 0/48 (0.0) | 0.0 | 0.0 | 0.0 – 0.0 |
| 11 | 8/26 (30.8) | 6.1 ± 14.9  (0.0 – 75.0) | 0.0 | 0.0 – 0.0 | 0/26 (0.0) | 0.0 | 0.0 | 0.0 – 0.0 |
| 12 | 18/27 (66.7) | 27.0 ± 33.4  (0.0 – 125.0) | 25.0 | 0.0 – 50.0 | 0/27 (0.0) | 0.0 | 0.0 | 0.0 – 0.0 |
| 13 | 18/77 (23.4) | 5.2 ± 12.7  (0.0 – 75.0) | 0.0 | 0.0 – 0.0 | 1/77 (1.3) | 0.0001 ± 0.002 (0.0 – 0.03) | 0.0 | 0.0 – 0.0 |
| 14 | 29/53 (54.7) | 16.3 ± 25.4  (0.0 – 100.0) | 0.0 | 0.0 – 25.0 | 0/53 (0.0) | 0.0 | 0.0 | 0.0 – 0.0 |
| Total | 352/922 (38.2) | 11.3 ± 22.8  (0.0 – 225.0) | 0.0 | 0.0 – 25.0 | 37/922 (4.0) | 0.01 ± 0.1  (0.0 – 1.15) | 0.0 | 0.0 – 0.0 |
| **Dataset 2 (DS2)** | | | | | | | | |
| 1 | 29/49 (59.2) | 2.3 ± 4.3  (0.0 – 20.0) | 2.0 | 0.0 – 4.0 | 0/49 (0.0) | 0.0 | 0.0 | 0.0 – 0.0 |
| 2 | 24/33 (72.7) | 2.4 ± 2.5  (0.0 – 10.0) | 2.0 | 0.0 – 2.0 | 0/33 (0.0) | 0.0 | 0.0 | 0.0 – 0.0 |
| 15 | 34/49 (69.4) | 4.6 ± 6.1  (0.0 – 22.0) | 2.0 | 0.0 – 4.0 | 0/49 (0.0) | 0.0 | 0.0 | 0.0 – 0.0 |
| 16 | 34/52 (65.4) | 3.7 ± 4.9  (0.0 – 26.0) | 2.0 | 0.0 – 6.0 | 0/52 (0.0) | 0.0 | 0.0 | 0.0 – 0.0 |
| 17 | 12/46 (26.1) | 1.9 ± 4.8  (0.0 – 24.0) | 0.0 | 0.0 – 2.0 | 0/46 (0.0) | 0.0 | 0.0 | 0.0 – 0.0 |
| 18 | 22/39 (56.4) | 3.1 ± 4.9  (0.0 – 20.0) | 2.0 | 0.0 – 4.0 | 0/39 (0.0) | 0.0 | 0.0 | 0.0 – 0.0 |
| 19 | 20/42 (47.6) | 2.2 ± 3.1  (0.0 – 12.0) | 0.0 | 0.0 – 4.0 | 0/42 (0.0) | 0.0 | 0.0 | 0.0 – 0.0 |
| 20 | 37/43 (86.1) | 8.1 ± 13.5  (0.0 – 84.0) | 4.0 | 2.0 – 8.0 | 0/43 (0.0) | 0.0 | 0.0 | 0.0 – 0.0 |
| 21 | 19/42 (45.2) | 2.1 ± 5.1  (0.0 – 32.0) | 0.0 | 0.0 – 2.0 | 0/42 (0.0) | 0.0 | 0.0 | 0.0 – 0.0 |
| 22 | 28/49 (57.1) | 6.1 ± 12.6  (0.0 – 52.0) | 2.0 | 0.0 – 4.0 | 0/49 (0.0) | 0.0 | 0.0 | 0.0 – 0.0 |
| 23 | 9/49 (20.0) | 0.5 ± 1.2  (0.0 – 6.0) | 0.0 | 0.0 – 0.0 | 0/49 (0.0) | 0.0 | 0.0 | 0.0 – 0.0 |
| 24 | 23/43 (53.5) | 5.4 ± 10.8  (0.0 – 54.0) | 2.0 | 0.0 – 6.0 | 0/43 (0.0) | 0.0 | 0.0 | 0.0 – 0.0 |
| 25 | 16/38 (42.1) | 2.3 ± 4.3  (0.0 – 20.0) | 0.0 | 0.0 – 2.0 | 0/38 (0.0) | 0.0 | 0.0 | 0.0 – 0.0 |
| 26 | 18/49 (36.7) | 3.2 ± 8.5  (0.0 – 54.0) | 0.0 | 0.0 – 4.0 | 0/49 (0.0) | 0.0 | 0.0 | 0.0 – 0.0 |
| 27 | 24/48 (52.1) | 3.3 ± 5.7  (0.0 – 32.0) | 2.0 | 0.0 – 4.0 | 0/48 (0.0) | 0.0 | 0.0 | 0.0 – 0.0 |
| Total | 349/667 (52.3) | 3.5 ± 7.3  (0.0 – 84.0) | 2.0 | 0.0 – 4.0 | 0/667 (0.0) | 0.0 | 0.0 | 0.0 – 0.0 |

Table S2: In-herd prevalence for *F. hepatica* and rumen flukes and mean egg count per gram faeces (EPG) among all herd samples (positives and negatives) with corresponding standard deviation (SD), range (minimum and maximum), median and interquartile range (25th to 75th percentile) in dataset 1 (DS1) and dataset 2 (DS2).

|  | ***F. hepatica*** | | | | **Rumen flukes** | | | |
| --- | --- | --- | --- | --- | --- | --- | --- | --- |
| **Herd (no.)** | Positive faecal samples (%) | Mean EPG ± SD (range) | Median | Interquartile range | Positive faecal samples (%) | Mean EPG ± SD (range) | Median | Interquartile range |
| **Dataset 1 (DS1)** | | | | | | | | |
| 1 | 44/81 (54.8) | 0.2 ± 0.3  (0.0 – 1.6) | 0.0 | 0.0 – 0.2 | 0/81 (0.0) | 0.0 | 0.0 | 0.0 – 0.0 |
| 2 | 2/47 (4.3) | 0.01 ± 0.05  (0.0 – 0.3) | 0.0 | 0.0 – 0.0 | 1/47 (2.1) | 0.02 ± 0.13  (0.0 – 0.9) | 0.0 | 0.0 – 0.0 |
| 3 | 0/55 (0.0) | 0.0 | 0.0 | 0.0 – 0.0 | 0/55 (0.0) | 0.0 | 0.0 | 0.0 – 0.0 |
| 4 | 1/66 (1.5) | 0.001 ± 0.02  (0.0 – 0.2) | 0.0 | 0.0 – 0.0 | 0/66 (0.0) | 0.0 | 0.0 | 0.0 – 0.0 |
| 5 | 0/196 (0.0) | 0.0 | 0.0 | 0.0 – 0.0 | 0/196 (0.0) | 0.0 | 0.0 | 0.0 – 0.0 |
| 6 | 0/52 (0.0) | 0.0 | 0.0 | 0.0 – 0.0 | 0/52 (0.0) | 0.0 | 0.0 | 0.0 – 0.0 |
| 7 | 12/68 (17.6) | 0.1 ± 0.4  (0.0 – 2.4) | 0.0 | 0.0 – 0.0 | 0/68 (0.0) | 0.0 | 0.0 | 0.0 – 0.0 |
| 8 | 0/44 (0.0) | 0.0 | 0.0 | 0.0 – 0.0 | 0/44 (0.0) | 0.0 | 0.0 | 0.0 – 0.0 |
| 9 | 46/82 (56.1) | 0.3 ± 0.7  (0.0 – 7.1) | 0.1 | 0.0 – 0.3 | 5/82 (6.1) | 0.01 ± 0.1  (0.0 – 0.5) | 0.0 | 0.0 – 0.0 |
| 10 | 6/48 (12.5) | 0.02 ± 0.1  (0.0 – 0.8) | 0.0 | 0.0 – 0.0 | 0/48 (0.0) | 0.0 | 0.0 | 0.0 – 0.0 |
| 11 | 0/26 (0.0) | 0.0 | 0.0 | 0.0 – 0.0 | 0/26 (0.0) | 0.0 | 0.0 | 0.0 – 0.0 |
| 12 | 19/27 (70.4) | 0.9 ± 1.6  (0.0 – 8.9) | 0.3 | 0.0 – 1.1 | 4/27 (14.8) | 0.03 ± 0.1  (0.0 – 0.7) | 0.0 | 0.0 – 0.0 |
| 13 | 0/77 (0.0) | 0.0 | 0.0 | 0.0 – 0.0 | 0/77 (0.0) | 0.0 | 0.0 | 0.0 – 0.0 |
| 14 | 0/53 (0.0) | 0.0 | 0.0 | 0.0 – 0.0 | 0/53 (0.0) | 0.0 | 0.0 | 0.0 – 0.0 |
| Total | 130/922 (14.1) | 0.07 ± 0.4  (0.0 – 8.9) | 0.0 | 0.0 – 0.0 | 10/922 (1.1) | 0.002 ± 0.04  (0.0 – 0.9) | 0.0 | 0.0 – 0.0 |
|  | | | | | | | | |
|  | | | | | | | | |
| **Dataset 2 (DS2)** | | | | | | | | |
| 1 | 7/49 (14.3) | 0.03 ± 0.1  (0.0 – 0.50) | 0.0 | 0.0 – 0.0 | 19/49 (38.8) | 0.3 ± 0.9  (0.0 – 4.80) | 0.0 | 0.0 – 0.2 |
| 2 | 2/33 (6.1) | 0.01 ± 0.1  (0.0 – 0.30) | 0.0 | 0.0 – 0.0 | 0/33 (0.0) | 0.0 | 0.0 | 0.0 – 0.0 |
| 15 | 18/49 (36.7) | 0.3 ± 0.6  (0.0 – 2.50) | 0.0 | 0.0 – 0.2 | 0/49 (0.0) | 0.0 | 0.0 | 0.0 – 0.0 |
| 16 | 4/52 (7.7) | 0.01 ± 0.03  (0.0 – 0.10) | 0.0 | 0.0 – 0.0 | 52/52 (100.0) | 33.8 ± 42.8  (0.3 – 192.7) | 18.5 | 7.5 – 41.6 |
| 17 | 6/46 (13.0) | 0.02 ± 0.1  (0.0 – 0.50) | 0.0 | 0.0 – 0.0 | 37/46 (80.4) | 5.8 ± 8.9  (0.0 – 30.0) | 0.8 | 0.1 – 7.4 |
| 18 | 5/39 (12.8) | 0.03 ± 0.1  (0.0 – 0.70) | 0.0 | 0.0 – 0.0 | 0/39 (0.0) | 0.0 | 0.0 | 0.0 – 0.0 |
| 19 | 1/42 (2.4) | 0.002 ± 0.02  (0.0 – 0.10) | 0.0 | 0.0 – 0.0 | 0/42 (0.0) | 0.0 | 0.0 | 0.0 – 0.0 |
| 20 | 15/43 (34.9) | 0.06 ± 0.1  (0.0 – 0.60) | 0.0 | 0.0 – 0.1 | 15/43 (34.9) | 0.1 ± 0.2  (0.0 – 1.0) | 0.0 | 0.0 – 0.1 |
| 21 | 10/42 (23.8) | 0.1 ± 0.3  (0.0 – 1.20) | 0.0 | 0.0 – 0.0 | 2/42 (4.8) | 0.01 ± 0.03  (0.0 – 0.20) | 0.0 | 0.0 – 0.0 |
| 22 | 0/49 (0.0) | 0.0 | 0.0 | 0.0 – 0.0 | 3/49 (6.1) | 0.01 ± 0.1  (0.0 – 0.40) | 0.0 | 0.0 – 0.0 |
| 23 | 1/45 (2.2) | 0.002 ± 0.01  (0.0 – 0.10) | 0.0 | 0.0 – 0.0 | 0/45 (0.0) | 0.0 | 0.0 | 0.0 – 0.0 |
| 24 | 10/43 (23.3) | 0.04 ± 0.1  (0.0 – 0.60) | 0.0 | 0.0 – 0.0 | 43/43 (100.0) | 58.0 ± 67.7  (1.1 – 292.4) | 35.0 | 15.6 – 80.3 |
| 25 | 4/38 (10.5) | 0.01 ± 0.03  (0.0 – 0.10) | 0.0 | 0.0 – 0.0 | 6/38 (15.8) | 0.02 ± 0.1  (0.0 – 0.20) | 0.0 | 0.0 – 0.0 |
| 26 | 0/49 (0.0) | 0.0 | 0.0 | 0.0 – 0.0 | 0/49 (0.0) | 0.0 | 0.0 | 0.0 – 0.0 |
| 27 | 6/48 (12.5) | 0.04 ± 0.1  (0.0 – 0.70) | 0.0 | 0.0 – 0.0 | 2/48 (4.2) | 0.07 ± 0.5  (0.0 – 3.10) | 0.0 | 0.0 – 0.0 |
| Total | 89/667 (13.4) | 0.04 ± 0.2  (0.0 – 2.50) | 0.0 | 0.0 – 0.0 | 179/667 (26.8) | 6.8 ± 26.4  (0.0 – 292.4) | 0.0 | 0.0 – 0.1 |
